# Progressive Pancytopenia in the Setting of Severe Pneumonia in a Patient With a History of Mantle Cell Lymphoma: A Case Report

**DOI:** 10.7759/cureus.116151

**Published:** 2026-09-12

**Authors:** Soshiant Raeesian, Sohrab R Mardani, Oscar Castillo, Srilekha Ravella, Luis Torres

**Affiliations:** 1 Medicine, Burrell College of Osteopathic Medicine, Las Cruces, USA; 2 Internal Medicine, Delray Medical Center, Delray Beach, USA

**Keywords:** hematologic malignancy, mantle cell lymphoma, neutropenia, pancytopenia, pneumonia

## Abstract

Progressive pancytopenia in a patient with a history of hematologic malignancy presents a diagnostic challenge because active hematologic disease and severe systemic illness can produce overlapping findings. We report a 74-year-old woman with previously treated mantle cell lymphoma who presented with fever, altered mental status, neutropenia, and multifocal pneumonia. During hospitalization, she developed rapidly progressive pancytopenia, prompting consideration of active hematologic disease, infection and critical illness, medication effects, and consumptive processes. Peripheral blood flow cytometry showed no immunophenotypic evidence of a lymphoproliferative disorder or acute leukemia. However, this finding did not establish the cause of the cytopenias. Her pneumonia progressed despite broad-spectrum antimicrobial therapy, and bronchoalveolar lavage cultures grew *Stenotrophomonas maltophilia*. She developed respiratory failure and shock requiring mechanical ventilation and vasopressor support, followed by refractory multiorgan failure. She died on hospital day 12. This case highlights the diagnostic challenge of rapidly progressive pancytopenia when severe pulmonary illness and a history of hematologic malignancy offer competing explanations. It emphasizes continual reassessment as serial hematologic and microbiologic findings reshape the differential diagnosis.

## Introduction

Pancytopenia has a broad differential diagnosis that includes impaired bone marrow production, marrow infiltration or replacement, nutritional deficiency, medication toxicity, infection, immune-mediated destruction, sequestration, and consumptive processes [1,2]. Determining the underlying cause can be particularly challenging in patients with a history of hematologic malignancy because active disease remains an important diagnostic consideration and a plausible explanation for worsening blood counts.

Prior treatment with rituximab may further complicate this assessment. Rituximab causes B cell depletion and has been associated with hypogammaglobulinemia, delayed immune reconstitution, late-onset neutropenia, and increased susceptibility to infection [3]. Severe infection and critical illness can also produce anemia, leukocyte abnormalities, thrombocytopenia, and activation of the coagulation system [4]. Progressive cytopenias in a previously treated patient with lymphoma therefore require consideration of both active hematologic disease and complications of acute systemic illness. Attribution to either process in isolation may be insufficient.

Pulmonary infection adds another layer of diagnostic uncertainty. Prior hematologic malignancy or immunosuppressive therapy may alter host defenses and increase susceptibility to both conventional and opportunistic pathogens. Diagnostic evaluation should therefore be guided by the underlying host factors and evolving clinical course [5]. We present a patient with previously treated mantle cell lymphoma who was neutropenic at admission and subsequently developed progressive anemia and thrombocytopenia during severe pneumonia. This evolving sequence created a diagnostic dilemma between active hematologic disease and systemic illness and illustrates how serial clinical, hematologic, and microbiologic findings can refine competing diagnoses even when a single definitive explanation is not established.

## Case presentation

A 74-year-old woman presented with fever, fatigue, progressive weakness, poor oral intake, and altered mental status. Her family reported approximately one week of high fevers, chills, night sweats, and increasing confusion before admission.

Her medical history was notable for mantle cell lymphoma previously treated with a rituximab-containing regimen followed by maintenance rituximab. The last documented maintenance dose occurred approximately one year before this hospitalization. Other relevant comorbidities included chronic obstructive pulmonary disease and heart failure with preserved ejection fraction.

Her cardiac history included prior transcatheter aortic valve replacement complicated by Achromobacter bacteremia and prosthetic valve endocarditis. She subsequently underwent redo sternotomy, removal of the transcatheter valve, and surgical aortic valve replacement with a tissue prosthesis in 2023. She also had a previous episode of Staphylococcus epidermidis bacteremia in 2022.

At presentation, her temperature was 39.4°C, and oxygen saturation was 92% on room air. Testing for coronavirus disease 2019 and influenza was negative. Lactate was 2.3 mmol/L. Initial blood cultures did not identify a pathogen.

Computed tomography of the chest demonstrated extensive right-sided pulmonary airspace consolidation, predominantly involving the right upper and lower lobes (Figure 1). The radiographic findings were consistent with multifocal pneumonia. Initial antimicrobial treatment consisted of cefepime and vancomycin. Persistent fever and early clinical deterioration prompted escalation to meropenem and micafungin while vancomycin was continued.

**Figure 1:**
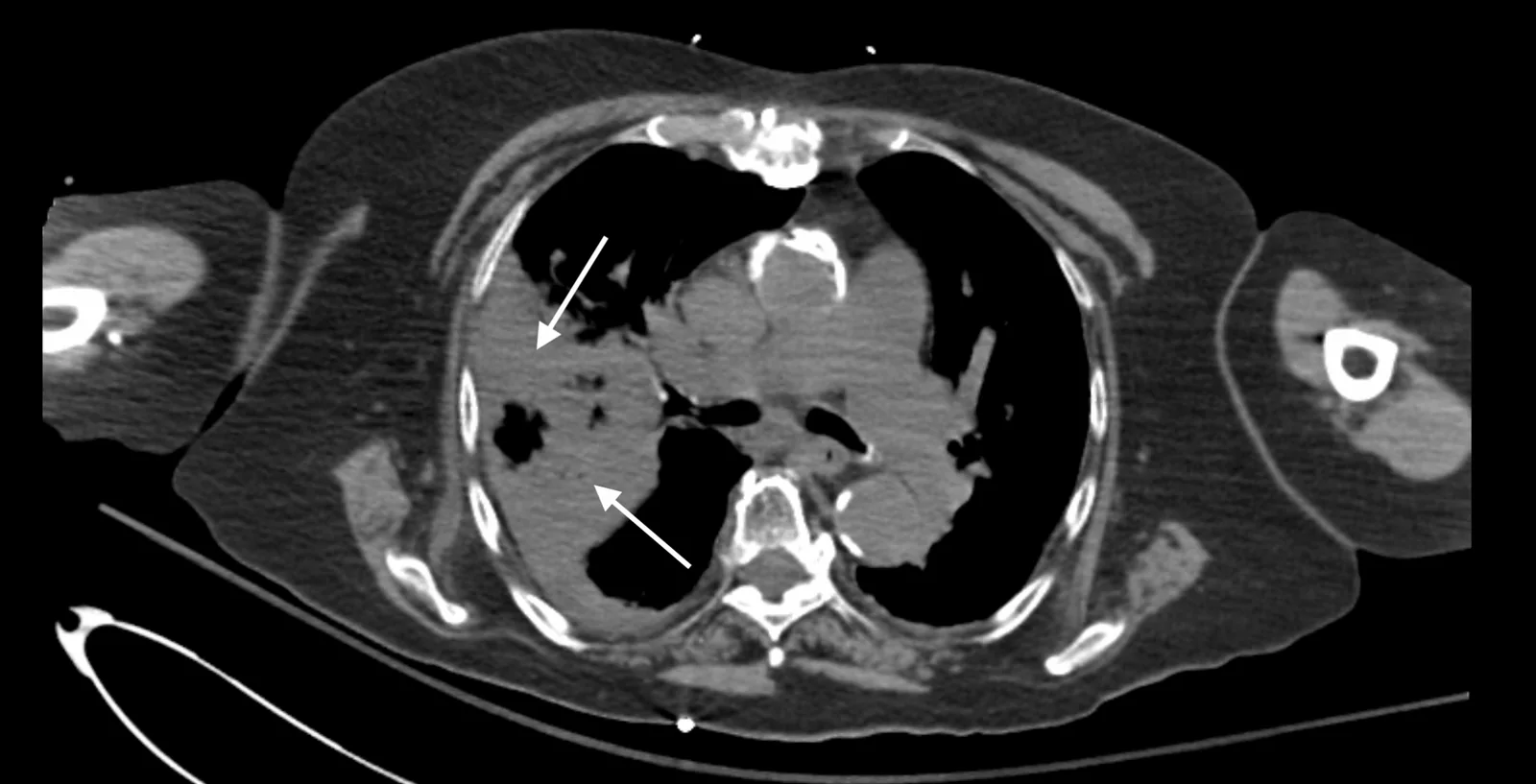
Axial computed tomography (CT) image of the chest demonstrating extensive right-sided pulmonary airspace consolidation, predominantly involving the right upper and lower lobes. White arrows indicate areas of dense airspace consolidation within the right lung.

The patient was leukopenic and neutropenic at admission, whereas her hemoglobin and platelet count were initially preserved. Her white blood cell (WBC) count was 1.6 × 10³/µL with an absolute neutrophil count (ANC) of 0.8 × 10³/µL. Hemoglobin was 12.3 g/dL, and the platelet count was 253 × 10³/µL.

Over the following days, the neutropenia became progressively more severe and was followed by substantial declines in hemoglobin and platelet count. By hospital day seven, the ANC had fallen to 0.2 × 10³/µL and fell to 0.1 × 10³/µL by hospital day 11. The platelet count decreased to 11 × 10³/µL by hospital day 12, while hemoglobin declined to 6.8 g/dL (Table 1).

**Table 1:** Hematologic laboratory trends during hospitalization. Serial white blood cell count, absolute neutrophil count (ANC), hemoglobin, and platelet values are shown by hospital day. ANC values were documented on hospital days 1, 4, 7, and 11; dashes indicate days on which an ANC value was not recorded.

Hospital Day	WBC (×10³/µL)	ANC (×10³/µL)	Hemoglobin (g/dL)	Platelets (×10³/µL)
Reference Range	4.0-11.0 (×10³/µL)	1.5-7.0 (×10³/µL)	12.0-16.0 (g/dL)	150-400 (×10³/µL)
Day 1	1.6	0.8	12.3	253
Day 2	0.7	-	10.3	193
Day 3	0.7	-	10.1	212
Day 4	0.6	0.4	9.7	209
Day 5	0.6	-	10.6	238
Day 6	0.5	-	10.7	224
Day 7	0.4	0.2	11.3	196
Day 8	0.2	-	9.9	120
Day 9	0.2	-	8.9	81
Day 10	0.2	-	7.7	51
Day 11	0.3	0.1	8.2	16
Day 12	0.2	-	6.8	11

The deterioration across all three blood cell lines prompted hematologic evaluation. Active mantle cell lymphoma, another acute hematologic process, reduced marrow production during severe systemic illness, medication effects, consumptive processes, and hemophagocytic lymphohistiocytosis (HLH) were among the considerations.

Additional abnormalities during the intensive care course included acute kidney injury, elevated lactate dehydrogenase, and markedly elevated ferritin. The reticulocyte percentage was low at 0.3%, consistent with a limited erythropoietic response to the worsening anemia. Fibrinogen was greater than 860 mg/dL, partial thromboplastin time was 42.7 seconds, and the international normalized ratio was 1.2. These findings did not establish a specific mechanism for the cytopenias.

Peripheral blood flow cytometry showed no abnormal blast population or immunophenotypic evidence of a B cell or T cell lymphoproliferative disorder. B cells were rare to absent. Specimen viability was limited, reducing the interpretability of the results.

The patient's pulmonary disease progressed despite antimicrobial therapy. On hospital day seven, she developed worsening acute hypoxic respiratory failure requiring endotracheal intubation and transfer to the intensive care unit. She subsequently developed shock requiring vasopressor support.

Bronchoscopy with bronchoalveolar lavage (BAL) was performed on hospital day eight because of progressive respiratory failure, persistent pulmonary infiltrates, and profound neutropenia. Flow cytometry of BAL material was limited by poor specimen viability and did not identify immunophenotypic evidence of a lymphoproliferative disorder on the limited panel performed. BAL and bronchial wash cultures subsequently grew Stenotrophomonas maltophilia. The isolate was reported as susceptible to trimethoprim sulfamethoxazole. Fungal and acid-fast bacillus cultures were negative.

Given her history of prosthetic valve infection and repeat aortic valve surgery, recurrent endocarditis was also considered. Transesophageal echocardiography performed on hospital day eight did not demonstrate a definite vegetation involving the prosthetic aortic valve. A linear echodensity along the aortic root was considered more consistent with postoperative or fibrinous material than active infection. Computed tomographic angiography did not identify a corresponding aortic root abnormality.

The patient's respiratory and hematologic abnormalities continued to worsen. Serial chest radiographs demonstrated increasing right basilar consolidation and a persistent right pleural effusion by early hospital day 12. She required continued mechanical ventilation, vasopressor support, and transfusion support for severe thrombocytopenia and anemia. Despite intensive supportive care, she developed refractory multiorgan failure and died on hospital day 12.

## Discussion

The temporal evolution of the pancytopenia was central to the diagnostic reasoning in this case. Neutropenia was present at admission, but the subsequent development of anemia and thrombocytopenia broadened the differential beyond active hematologic disease to include impaired marrow production during systemic illness, consumptive processes, and effects of treatment [1,2,4].

The low reticulocyte percentage of 0.3% indicated a limited erythropoietic response but did not identify the primary mechanism of anemia [1,2]. The rapid decline in platelet count also raised concern for consumptive and coagulation-related processes. Thrombocytopenia is common in sepsis and may result from altered platelet production, activation and consumption, immune mechanisms, or disseminated intravascular coagulation (DIC) [6]. Fibrinogen is an acute phase reactant and may remain normal or elevated during sepsis despite ongoing coagulation activation and consumption [7]. Therefore, the markedly elevated fibrinogen did not exclude sepsis-associated coagulopathy, although the available coagulation studies were insufficient to establish overt DIC as the principal explanation for the cytopenias.

Medication effects, including recent antimicrobial exposure, also remained a consideration, but the available temporal data did not identify a specific agent as the cause of the cytopenias.

HLH was considered because infection and malignancy are recognized triggers and the syndrome may resemble severe sepsis or multiorgan dysfunction [8]. Progressive cytopenias and marked hyperferritinemia supported its consideration. However, the available clinical information was insufficient to establish that accepted diagnostic criteria were fulfilled, and HLH remained unconfirmed.

Active mantle cell lymphoma also remained an important consideration. Peripheral blood flow cytometry did not show an abnormal blast population or circulating lymphoproliferative disorder, but interpretation was limited by specimen viability and the study did not directly assess the bone marrow [9]. Bone marrow examination was not performed in the setting of the patient’s rapid clinical deterioration, and marrow involvement therefore remained unresolved.

The pulmonary course created an additional source of diagnostic uncertainty. S. maltophilia is an opportunistic Gram-negative pathogen associated with substantial comorbidity, prior broad-spectrum antimicrobial exposure, invasive devices, immunosuppression, and critical illness [10,11], several of which were present in this patient. Pneumonia was present at admission, whereas BAL was obtained on hospital day eight after several days of broad-spectrum antimicrobial therapy and after respiratory failure required intubation. Isolation of S. maltophilia from BAL and bronchial wash cultures confirmed its presence in the lower respiratory tract but could not distinguish with certainty among an initial pulmonary pathogen, a secondary infection acquired during hospitalization, or respiratory colonization [10-12].

S. maltophilia possesses intrinsic resistance mechanisms, including L1 and L2 beta-lactamases, and carbapenems do not provide reliable activity against the organism [10,12]. Consequently, deterioration during meropenem therapy did not argue against clinically significant S. maltophilia infection. The isolate was susceptible to trimethoprim sulfamethoxazole, but the available record did not allow reliable reconstruction of the timing of organism identification, susceptibility reporting, and directed therapy. The effect of earlier targeted treatment therefore cannot be determined.

In this case, BAL provided lower respiratory microbiologic information that had not been obtained from the initial blood cultures. Its diagnostic contribution must nevertheless be interpreted in the context of the late timing of sampling and preceding antimicrobial exposure [13].

Previous rituximab exposure remained relevant, but its contribution to the presenting neutropenia or immune vulnerability could not be established [3]. Peripheral flow cytometry showed rare to absent B cells, but profound leukopenia and limited specimen viability reduced the interpretability of this finding as an assessment of immune reconstitution. Immunoglobulin concentrations and serial B cell measurements were unavailable.

Given her history of prosthetic valve endocarditis, recurrent infection also remained a consideration despite negative routine blood cultures because blood culture-negative endocarditis is recognized [14]. However, transesophageal echocardiography showed no definite prosthetic vegetation, the aortic root echodensity was considered more consistent with postoperative or fibrinous material, and computed tomographic angiography showed no corresponding aortic root abnormality. Together, these findings made recurrent endocarditis a less compelling explanation as the pulmonary process progressed.

The principal limitation of this case is that no single mechanism could be established for the progressive pancytopenia, and the late timing of respiratory sampling prevented definitive attribution of S. maltophilia to the pneumonia present at admission. Plausible contributors to the cytopenias included impaired marrow production from systemic illness or active hematologic disease, consumptive processes associated with infection, medication effects, and prior therapy. The educational value of the case lies in demonstrating how serial hematologic, microbiologic, imaging, and clinical findings can shift the relative likelihood of competing diagnoses even when a definitive explanation cannot be established.

## Conclusions

Rapidly progressive pancytopenia in a patient with a history of hematologic malignancy and severe infection requires simultaneous consideration of active hematologic disease and nonmalignant systemic causes. In this case, the evolution from neutropenia to pancytopenia, nondiagnostic peripheral blood flow cytometry, and later recovery of Stenotrophomonas maltophilia from BAL narrowed but did not resolve the competing explanations. No single process definitively accounted for the cytopenias. Serial reassessment of hematologic trends, microbiologic findings, and the overall clinical trajectory is essential when the differential remains dynamic. When uncertainty persists, further diagnostic testing should be guided by clinical stability, expected diagnostic yield, and whether the result would meaningfully change management.
